# Results of multigene panel testing in familial cancer cases without genetic cause demonstrated by single gene testing

**DOI:** 10.1038/s41598-019-54517-z

**Published:** 2019-12-06

**Authors:** Mev Dominguez-Valentin, Sigve Nakken, Hélène Tubeuf, Daniel Vodak, Per Olaf Ekstrøm, Anke M. Nissen, Monika Morak, Elke Holinski-Feder, Arild Holth, Gabriel Capella, Ben Davidson, D. Gareth Evans, Alexandra Martins, Pål Møller, Eivind Hovig

**Affiliations:** 1 Department of Tumor Biology, Institute for Cancer Research, Oslo University Hospital, Oslo, Norway; 2 Inserm-U1245, UNIROUEN, Normandie Univ, Normandy Centre for Genomic and Personalized Medicine, Rouen, France; 3 Interactive Biosoftware, Rouen, France; 4 Medizinische Klinik und Poliklinik IV, Campus Innenstadt, Klinikum der Universität München, Ziemssenstr. 1, Munich, Germany; 5 MGZ—Medizinisch Genetisches Zentrum, Munich, Germany; 6 Department of Pathology, Oslo University Hospital, Norwegian Radium Hospital, Oslo, Norway; 7 Hereditary Cancer Program, Catalan Institute of Oncology, Insititut d’Investigació Biomèdica de Bellvitge (IDIBELL), ONCOBELL Program, L’Hospitalet de Llobregat, Barcelona, Spain, and Centro de Investigación Biomédica en Red de Cáncer (CIBERONC), Barcelona, Spain; 8 University of Oslo, Faculty of Medicine, Institute of Clinical Medicine, N-, 0316 Oslo, Norway; 9 Department of Genomic Medicine, Division of Evolution and Genomic Sciences, The University of Manchester, Manchester Academic Health Science Centre, St. Mary’s Hospital, Manchester, United Kingdom; 10 Prevent Breast Cancer Centre, Wythenshawe Hospital, Southmoor Road, Manchester, United Kingdom; 11 Department of Human Medicine, Universität Witten/Herdecke, Wuppertal, Germany; 12 Department of Informatics, University of Oslo, Oslo, Norway; 13 Centre for Cancer Cell Reprogramming, Institute of Clinical Medicine, Faculty of Medicine, University of Oslo, Oslo, Norway

**Keywords:** Diagnostic markers, Predictive markers

## Abstract

We have surveyed 191 prospectively sampled familial cancer patients with no previously detected pathogenic variant in the *BRCA1/2*, *PTEN*, *TP53* or DNA mismatch repair genes. In all, 138 breast cancer (BC) cases, 34 colorectal cancer (CRC) and 19 multiple early-onset cancers were included. A panel of 44 cancer-predisposing genes identified 5% (9/191) pathogenic or likely pathogenic variants and 87 variants of uncertain significance (VUS). Pathogenic or likely pathogenic variants were identified mostly in familial BC individuals (7/9) and were located in 5 genes: *ATM* (3), *BRCA2* (1), *CHEK2* (1), *MSH6* (1) and *MUTYH* (1), followed by multiple early-onset (2/9) individuals, affecting the *CHEK2* and *ATM* genes. Eleven of the 87 VUS were tested, and 4/11 were found to have an impact on splicing by using a minigene splicing assay. We here report for the first time the splicing anomalies using this assay for the variants *ATM* c.3806A > G and *BUB1* c.677C > T, whereas *CHEK1* c.61G > A did not result in any detectable splicing anomaly. Our study confirms the presence of pathogenic or likely pathogenic variants in genes that are not routinely tested in the context of the above-mentioned clinical phenotypes. Interestingly, more than half of the pathogenic germline variants were found in the moderately penetrant *ATM* and *CHEK2* genes, where only truncating variants from these genes are recommended to be reported in clinical genetic testing practice.

## Introduction

Based on twin studies and recent reports, 30–45% of colorectal cancer (CRC) cases may involve a heritable component^1–4^. However, highly-penetrant pathogenic germline variants that explain familial aggregation and/or an early-onset of the disease (e.g. pathogenic variants in *APC* and DNA mismatch repair (MMR) genes) are detected in only 5–10% of the cases^5^. In contrast, in hereditary breast and ovarian cancer (HBOC), up to ~25% of the cases can be explained by the highly penetrant risk genes *BRCA1* and *BRCA2*^6^.

Next-generation sequencing (NGS) studies have reported that as many as ~18% of patients diagnosed with CRC < age of 50 years have pathogenic germline variants in genes that are not traditionally associated with CRC, including *ATM*, *CHEK2*, *BRCA1*, *BRCA2*, *CDKN2A* or *PALB2*^7,8^, while up to 15% of HBOC patients harbor pathogenic variants in known BC predisposing genes, including *RAD51C*, *RAD51D*, *ATM*, *CHEK2*, *BRIP1*, *PALB2*, *BARD1*, *RECQL*, *TP53*, *CDH1* and *NBN*^9,10^. Hence, in approximately 60%-70% of all tested hereditary CRC or HBOC patients, the genetic predisposition is still unknown. There is a need to determine whether these variants contribute to hereditary CRC or HBOC risk via the combination of low- and moderate-penetrance susceptibility alleles, or in conjunction with environmental factors^7,8,11–13^

Importantly, phenotype-driven panels provide a notable rate of detection of pathogenic variants with clinical actionability. NGS-based panel screening described 8–13% of pathogenic variants in *BRCA1/2* negative HBOC patients^14,15^. Therefore, there are new genes recently discovered by the use of gene panel testing for which there is still limited data regarding the degree of their association to cancer risk, and the medical management guidelines are scant or non-existent for some of them^16–18^.

A relatively common event that complicates the interpretation of gene test results is the detection of variants of unknown significance (VUS). Regarding *BRCA1* and *BRCA2* genes, up to 20% of all variants are still being classified as VUS^19^, while about one-fifth to one-third are classified in the case of the DNA MMR gene variants^20^.

Because multiple cancer gene panel testing is rapidly replacing sequential single-gene testing, we need to know how to improve in the way we interpret the findings from panel testing patients in cancer kindreds who have been previously tested for the *BRCA1/2*, *PTEN*, *TP53* and MMR genes without detection of pathogenic variants. The goals were to gain information on to which degree other genes may have been causative for cancer in the patients and their relatives, and to be informed on how such genes were deranged to discriminate between normal and disease-causing variants. In addition, we analyzed the impact of a subset of VUS on RNA splicing by the use of minigene assays.

## Methods

The methods were performed in accordance with the relevant guidelines and regulations.

### Study population

The study population was selected from the Hereditary Cancer Biobank (n = 161), which is part of the out-patient inherited cancer clinic from the Norwegian Radium Hospital (Norway)^21^ and the Department of Genomic Medicine (n = 30) from the University of Manchester (United Kingdom)^12^.

The selection criteria for the 191 individuals were as follows:

- Being member of cancer family or having a personal history of cancer;
- Presence of multiple early-onset cancer at early age of onset, including BC, gynecological cancer, CRC, or thyroid cancer;
- Familial CRC cases: Families that fulfilled the Amsterdam criteria or the revised Bethesda guidelines who had tested negative for pathogenic variants in the mismatch repair (MMR) genes;
- Familial BC cases: Women with BC or gynecological cancer who had tested negative for pathogenic *BRCA1* or *BRCA2* variants.

Overall, our study subjects (n = 191) were demonstrated not to carry pathogenic variants in *BRCA1/2*, *TP53*, *PTEN* or MMR genes, or large exon deletions/duplications involving these genes by standard diagnostics.

**Figure 1:**
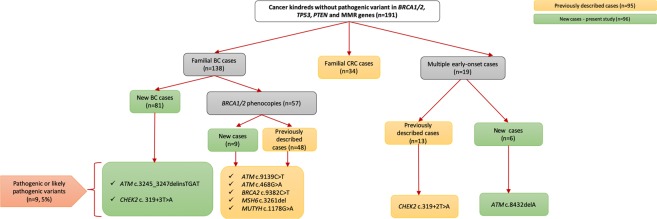
Relational flowchart of the cancer kindreds and results from the study.

In all, 138 breast cancer (BC) cases were included, where 57 were denoted as *BRCA* phenocopies (cases who were tested negative for their respective family’s *path_BRCA1* (n = 18) and *path_BRCA2* variants (n = 39)). Thirty-four CRC and 19 multiple early-onset cancers cases were also included (Fig. 1). Out of the 191 familial cancer patients, we have previously reported on 95 individuals (48 phenocopies, 34 familial CRC and 13 multiple early-onset cases)^12,22,23^. The current study added 96 new cases, including 81 familial BC cases, 9 phenocopies and 6 additional subjects having multiple early-onset cancer (Fig. 1).

Ethical approval for the study was granted by the Norwegian Data Inspectorate (ref. 2001/2988-2) and Ethical Review Board (ref. S-02030). All examined patients signed an informed consent for their participation in the study.

### Targeted sequencing and data analysis

Genomic DNA was isolated from peripheral blood samples and a 44-gene panel targeted sequencing was carried out using a TruSeq amplicon-based assay v.1.5 on a MiSeq apparatus (Supplementary Table 1)^12,22,23^. The mean depth of sequencing coverage ranged from 124 to 535, and the fraction of target bases with coverage ≥25 ranged from 80% to 94%.

Sequence alignments with paired-end sequence reads to the human reference genome (build GRCh37) were obtained through the BWA-mem algorithm (v.0.7.8-r55), where sorting and indexing procedures were undertaken with Samtools (v.1.1). Genotyping of single nucleotide variants (SNV) and short indels, as well as quality filtering of the raw genotype calls, were done according to GATK’s best practice procedures, as described previously in more detail^12,22,23^.

Functional annotation of the variants was performed with ANNOVAR (version November 2015)^24^ and using five databases (gnomAD, dbSNP (build 147)^25^, 1000 Genome Project phase3^26^, UniProt (release March 2016)^27^, and Pfam protein domain (v29, December 2015)^12,22,23,28^.

### Nomenclature and classification of genetic variants

The Human Genome Variation Society (HGVS) guidelines were used to describe the detected genetic variants^29^. The following databases: Evidence-based Network for the Interpretation of Germline Mutant Alleles (ENIGMA), Breast Cancer Information Core (BIC), International Society of Gastrointestinal Hereditary Tumors (InSiGHT), Leiden Open Variation Database (LOVD), ClinVar, and the Human Gene Mutation Database (HGMD) were interrogated for the clinical significance of the identified genetic variants (in their releases as of August 2018).

We applied the American College of Medical Genetics and Genomics (ACMG) guidelines for the classification and interpretation of the clinical significance of the variants^30^. In addition, when a novel variant was identified in the study, we considered it to be pathogenic or likely pathogenic if either one of the following criteria were met: a) introduction of a premature stop codon in the protein coding sequence (nonsense or frameshift); b) occurrence at positions +1/+ 2 or −1/−2 of donor or acceptor splice sites, respectively; or c) whole-exon deletions.

### Validation by cycling temperature capillary electrophoresis

Cycling temperature capillary electrophoresis was used to validate the 13 pathogenic variants found in the study. The method has been previously described^12,31–33^. The variant melting profile tool (http://meltprimer.ous-research.no/)^34^ was used to design the amplicons. Details about the primer sequences, PCR reaction conditions and electrophoresis settings are available upon request.

### *In silico* analyses of VUS

The MaxEntScan (MES) and SSF-like (SSFL) methods were used to predict variant-induced alterations in 3′ and 5′ splice site (3′ss and 5′ss) strength, as described by Houdayer *et al*. 2012^35^, but using the integrated software tool Alamut Batch version 1.5 (Interactive Biosoftware, France). As previously described by Soukarieh *et al*.^36^ and in prior studies^12,22,23^, we considered:

- If ΔMES ≥ 15% and ΔSSFL ≥ 5%: the variant mapping at a splice site could negatively impact exon inclusion^35^;
- If negative Δ scores were provided by at least 2 out of the 3 exonic splicing regulatory elements (ESR)-dedicated *in silico* tools: the variant located outside the splice sites was considered as a probable inducer of exon skipping^36^.

The Align-GVGD (the VUS were predicted as deleterious when the values were C35 or higher), SIFT, MAPP, PolyPhen-2 and MutationTaster^12,22,23,37–41^ were used to predict the protein impact of missense variants. The Alamut Batch version 1.4.4 (Interactive Biosoftware, http://www.interactive-biosoftware.com) was used for the protein *in silico* predictions.

### Cell-based minigene splicing assay

We performed functional assays based on comparative analysis of the splicing pattern of wild type (WT) and mutant reporter minigenes to analyze the impact on splicing of each selected variant, as previously reported^12,22,23^.

The genomic regions containing the exon of interest and ~150 nucleotides of the flanking introns were amplified by PCR using patients’ DNA as template and primers (Supplementary Table 2). The PCR-amplified fragments were inserted into a previously linearized pCAS2 vector, generating the representative minigenes. The inserts of all clones were sequenced and the WT and mutant constructs were transfected in parallel into HeLa cells grown at ~70% confluence. The total RNA was extracted after 24 h, and the transcripts were analyzed by semi-quantitative RT-PCR (Supplementary Table 2). The RT-PCR products were separated by electrophoresis, followed by gel-purification. Sanger sequencing was performed for the proper identification of the minigene transcripts. Finally, fluorescent RT-PCR reactions followed by capillary electrophoresis were performed to quantify the splicing events. The computational analysis was performed using GeneMapper v5.0 software (Applied Biosystems).

### Immunohistochemistry of ATM c.3806A > G carrier

We assessed *in situ* the effect of *ATM* c.3806A > G p.(Lys1269Arg) at the protein level by performing an immunohistochemistry (IHC) analysis. Paraffin-embedded material from the patient tumor was immuno-stained for the ATM protein: Briefly, 4-µm sections were placed on SuperFrost^®^ Plus microscope slides. Antigen retrieval was performed in a Dako PT Link in Dako’s pH 6 (Low pH) retrieval solution according to the Dako’s Flex protocol and stained manually. We used the anti-ATM antibody clone 2C1 (Abcam ab78, dilution 1:2000). The ATM protein expression was assessed either as retained (normal), absent, or weak (i.e. tumor cell staining intensity was reduced compared with that of the normal internal control).

## Results

### Pathogenic or likely pathogenic germline findings

We utilized a 44-cancer gene panel in 191 high-risk individuals in order to identify other high- or moderate- penetrance genes that could be implicated in the genetic cause of these phenotypes (Supplementary Table 1). In the combined cohort, we identified 9 cases (5% of 191) who carried 9 pathogenic or likely pathogenic variants that were confirmed by cycling temperature capillary electrophoresis, showing 100% concordance (Fig. 1, Table 1).

**Table 1:** Description of the 9 pathogenic or likely pathogenic variants found in phenocopies, familial BC and multiple early-onset cancers cases analyzed by 44-cancer gene panel testing.

Genetic Variant	Penetrance of the gene*	Cancer type	Classification based on the described databases and ACMG	Reported?
*ATM* (NM_000051) c. 9139C > T (p.Arg3047Ter)	Moderate-penetrance	Phenocopies	Pathogenic, Likely Pathogenic/Pathogenic	Dominguez-Valentin *et al*., 2018
*ATM* (NM_000051) c.3245_3247delinsTGAT (p.His1082Leufs)	Moderate-penetrance	Familial BC	Pathogenic/Pathogenic	Current study
*ATM* (NM_000051) c.468G > A (p.Trp156*)	Moderate-penetrance	Phenocopies	Not described/Pathogenic	Dominguez-Valentin *et al*., 2018
*ATM* (NM_000051) c.8432delA (p.Lys2811SerfsTer46)	Moderate-penetrance	Multiple early onset	Pathogenic/Pathogenic	Current study
*BRCA2* (NM_000059) c.9382C > T (p.Arg3128Ter)	High-penetrance	Phenocopies	Pathogenic/Pathogenic	Dominguez-Valentin *et al*., 2018
*CHEK2* (NM_007194) c.319 + 2 T > A	Moderate-penetrance	Multiple early onset	Likely Pathogenic/Pathogenic	Dominguez-Valentin *et al*., 2017
*CHEK2* (NM_007194) c.319 + 2T > A	Moderate-penetrance	Familial BC	Likely Pathogenic/Pathogenic	Current study
*MSH6* (NM_000179) c.3261del (p.Phe1088SerfsTer2)	High-penetrance	Phenocopies	Pathogenic/Pathogenic	Dominguez-Valentin *et al*., 2018
*MUTYH* (NM_012222) c.1178G > A (p.Gly393Asp)	Recessive risk	Phenocopies	Likely Pathogenic/Uncertain significance	Dominguez-Valentin *et al*., 2018

BC: breast cancer; ACMG: American College of Medical Genetics and Genomics, *Dominguez-Valentin *et al*.^22^.

### From high- to moderate-penetrance genes identified in the study

Two pathogenic variants (2/191, 1%) were identified in high-penetrance genes, namely *BRCA2* c.9382C > T p.(Arg3128*) (this variant was previously undetected, as only the *path_BRCA2* c.6591_6592delTG was demonstrated in a relative subjected to previous testing) and *MSH6* c.3261delC (p.Phe1088Serfs*2) from two phenocopy individuals.

**Figure 2:**
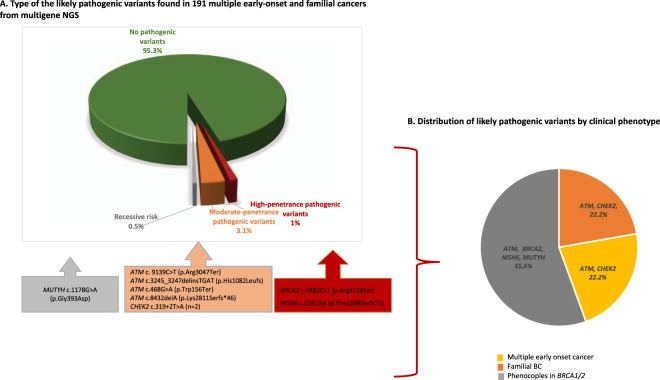
Overview of the germline variants found in the study. (**A**) Type of the pathogenic or likely pathogenic variants found in 191 multiple early-onset and familial cancers from multigene NGS. (**B**). Distribution of pathogenic or likely pathogenic variants by clinical phenotype

In the case of the heterozygous *MUTYH* c.1178G > A (p.Gly393Asp), we reported this gene as a recessive risk and it was found in a phenocopy individual, as previously reported by us^12^ (Table 1, Fig. 2A).

Six pathogenic variants (6/191, 3.1%) were detected in moderate-penetrance genes and included: *ATM* c.9139C > T (p.Arg3047*) and *ATM* c.468G > A (p.Trp156*) found in two phenocopies cases^12^, the Norwegian founder *ATM* c.3245_3247delinsTGAT (p.His1082Leufs)^42^, and the *ATM* c.8432delA (p.Lys2811Serfs*46) found in familial BC and multiple early-onset case, respectively. In addition, we identified *CHEK2* c.319 + 2T > A (IVS2 + 2T > A in multiple early-onset and familial BC cases (Fig. 2A).

### Pathogenic or likely pathogenic variants by clinical phenotype

When analyzed by clinical phenotype, we described that phenocopies and familial BC patients are the most affected cohorts by the presence of pathogenic variants in high- and moderate-penetrance genes, respectively. In detail, phenocopies were affected by the presence of pathogenic variants in the high-penetrance genes *BRCA2* and *MSH6*, the moderate-penetrance gene: *ATM* and *MUTYH* gene. Familial BC cases were predominately affected by pathogenic or likely pathogenic variants in moderate-penetrance (*ATM*, *CHEK2*) genes (Fig. 2B).

### *In silico* and minigene analysis of VUS

In addition to the 9 pathogenic variants found in the study, the NGS results revealed the presence of 87 variants that we interpreted as VUS. When *in silico* splicing and protein predictions were applied, 17 VUS in 15 genes were selected as more likely to be functionally relevant: *APC*, *ATM*, *AXIN2*, *BRCA2*, *BUB1*, *CDH1*, *CHEK1*, *CHEK2*, *MAP3K1*, *MSH2*, *NOTCH3*, *POLE*, *RAD51B*, *RAD51D*, and *STK11* (Supplementary Table 3). The MAF of these variants in the gnomAD database were very low (<1%) or not reported (Supplementary Table 3). Given that patient RNA was not available, we decided to experimentally assess the impact on splicing of the 17 VUS by performing cell-based minigene assays. Because of technical difficulties, 6 variants were excluded from the analysis (Supplementary Table 3).

**Figure 3:**
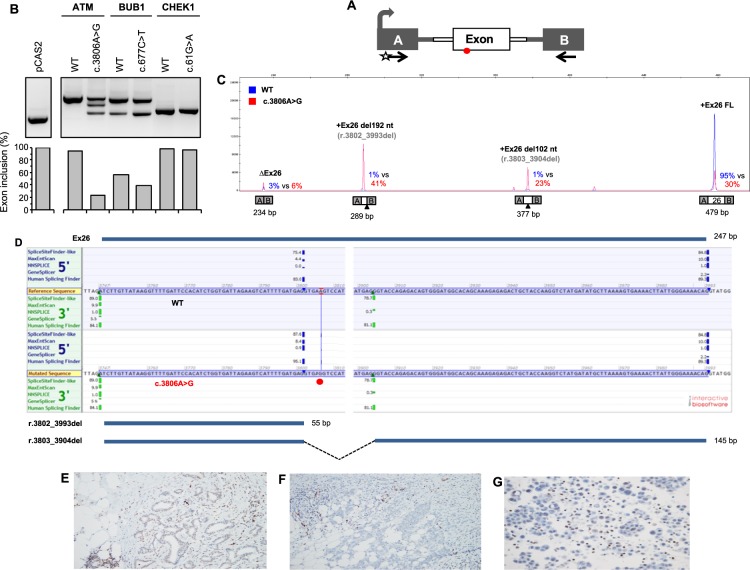
Detection of variant-induced splicing alterations by using a minigene splicing assay. (**A)** Structure of pCAS2 minigenes used in this assay. The gray arrow indicates the CMV promoter, boxes represent exons, lines in between indicate introns, and arrows below the exons represent primers used in RT-PCR reactions. The start indicates a fluorescent primer. (**B**) Analysis of the splicing pattern of the pCAS2-derived minigenes carrying *ATM*, *BUB1* and *CHEK1* variants as indicated. The minigene assays were performed as described under Materials and Methods. The top panel shows the RT-PCR products obtained for WT and mutant constructs separated by electrophoresis on an ethidium bromide–stained 2.5% agarose gel. The bottom panel shows the quantification of the RT-PCR products focusing on the relative level of exon inclusion (i.e. products corresponding to full-length minigene transcripts, FL). Quantification was performed after separating the fluorescent RT-PCR products by capillary electrophoresis on an automated sequencer. as described under Materials and Methods. (**C)** Representative fluorescent RT-PCR experiment using pCAS2 minigenes carrying *ATM* exon 26 (WT and c.3806A > G, as indicated). The panel shows superposed peaks corresponding to the WT and mutant RT-PCR products (in blue and red, respectively). Splicing events are expressed as % of the total amount of RT-PCR products obtained for each WT and mutant minigene construct (mutant vs WT). Results are representative of two independent experiments. (**D**) Splice sites-dedicated bioinformatics predictions and representation of the major splicing events detected in the minigene assay relative to *ATM* exon 26. The position of c.3806A > G is indicated by the red circle. *In silico* predictions of splice sites were obtained by simultaneously interrogating 5 algorithms (SpliceSiteFinder-Like, MaxEntScan, NNSPLICE, GeneSplicer and Human Splice Finder) through the integrated software Alamut Visual Version 2.10 (Interactive Biosoftware, France). For simplicity, only scores relative to *ATM* 5′ss and 3′ss (natural, *de novo* or cryptic sites) detected in the minigene assay are shown. The properly spliced full-length exon (FL) is represented above the panel whereas the aberrantly spliced truncated versions of the exon are indicated underneath. (**E**) Immunohistochemical staining for ATM in the patient’s tumor. (**F**) Breast carcinoma control. **(G)** Malignant effusions from other cancer cases. Host cells, consisting of lymphocytes, macrophages, stromal cells and adipocytes, are strongly positive. The tumor was immunostained for the ATM protein as follows. Briefly, 4-µm sections were placed on SuperFrost^®^ Plus microscope slides. Antigen retrieval was performed in a Dako PT Link in Dako’s pH 6 (Low pH) retrieval solution according to the Dako´s Flex protocol and stained manually. We used the anti-ATM antibody clone 2C1 (Abcam ab78, dilution 1:2000). The ATM protein expression was assessed as retained (normal), absent, or weak (i.e. tumor cell staining intensity was reduced compared with that of the normal internal control).

An overview of the results for the remaining 11 VUS is shown in Supplementary Table 3, which includes data for 8 variants that we have published elsewhere. More precisely, we previously reported that *APC* c.721G > A, *BRCA2* c.116C > T, *CDH1* c.1774G > T, *CHEK2* c.608A > G *MAP3K1* c.764A > G and *NOTCH3* c.5854G > A did not affect the splicing pattern of the minigene transcripts^12,22^, whereas *MSH2* c.815C > T and *NOTCH3* c.1490C > T were previously described to induce partial skipping of exons 5 and 9, respectively^22,43^. Here, we report that *ATM* c.3806A > G induces a complex pattern of aberrant splicing as described below (Fig. 3A–D). Moreover, we found that *BUB1* c.677C > T causes a moderate increase in exon skipping, whereas *CHEK1* c.61G > A has no major effect in the context of our minigene assay (Fig. 3B).

### ATM c.3806A > G, BUB1 c.677C > T and CHEK1 c.61G > A

In the *ATM* gene, we identified a transition A > G at position c.3806 in the exon 26 (c.3806A > G, p.(Lys1269Arg), rs146017595) in a woman with a BC diagnosis at 46 years. The pedigree indicated a family history of BC, ovarian cancer and colon cancer, including a mother with BC diagnosis at 56 years, and two maternal aunts with BC diagnoses at 43 and 46 years, respectively (data not shown). To our knowledge, *ATM* c.3806A > G (p.Lys1269Arg) has not been classified in the literature either as pathogenic or benign. The variant is present in the general population at a very low allele frequency according to the gnomAD database (6.668 e-05) and is reported in ClinVar as a VUS (Variation ID: 185981). RNA splicing-dedicated *in silico* analyses predicted that this variant may affect RNA maturation by modifying potential splicing signals within *ATM* exon 26 (Supplementary Table 3). Our minigene assay results, shown in Fig. 3, revealed that *ATM* c.3806A > G indeed causes aberrant splicing, leading to a decrease in full-length transcripts (from 95% to 23%). Two main aberrant isoforms were detected in this assay: one presenting a terminal deletion of 192 nucleotides (corresponding to *ATM* r.3802_3993del, p.Val1268_Glu1331del) and the other presenting an internal deletion of 102 nucleotides within *ATM* exon 26 (corresponding to *ATM* r.3803_3904del, p.Val1268_1301del). Both alterations result from the usage of a new splice site directly introduced by the variant as predicted by the MaxEntScan algorithm (Supplementary Table 3). The terminal deletion indicates that the new 5′ ss can, in some transcripts, outcompete the natural 5′ss of exon 26 and be spliced to the following exon, whereas the internal deletion shows that *ATM* c.3806A > G also leads, in part, to the activation of a cryptic 3′ss downstream in exon 26 (Fig. 3D). Protein-based *in silico* analyses were inconsistent regarding the effect that *ATM* c.3806A > G (p.Lys1269Arg) may have on protein structure and function (Supplementary Table 3). We assessed *in situ* the effect at the protein level of *ATM* c.3806A > G by IHC analysis. The results revealed a weak-to-absent staining in the proband’s tumor as compared to normal surrounding tissue (Fig. 3E–G), also suggesting that the germline *ATM* c.3806A > G may be implicated in tumor development.

The *BUB1* c.677C > T variant was identified in a female with a BC diagnosis at 56 years who did not carry any pathogenic variant in *BRCA1/2* or MMR genes. In addition to the *BUB1* variant, the patient carried two class 1/2 variants: *NOTCH3* c.1487C > T (p.Pro496Leu) and *NOTCH3* c.3704A > T (p.His1235Leu). The *BUB1* variant is present in the general population at a low allele frequency according to the gnomAD database (1.999 e-3), and has not been reported in ClinVar. As *ATM* c.3806A > G, the protein-based *in silico* analyses were inconsistent regarding the effect on protein structure and function for *BUB1* c.677C > T (Supplementary Table 3). The minigene assay showed a moderate increase in exon skipping (43% to 61%) (Fig. 3B).

Finally, we identified a *CHEK1* c.61G > A in a *BRCA2* phenocopy case, which is currently not reported in ClinVar, and the minigene assay showed no major splicing defect, which is in contrast to the *in silico* predictions (Fig. 3B).

## Discussion

When applying multigene panel testing to a set of 191 familial cancer cases, we identified 9 cases (5%) harboring pathogenic or likely pathogenic variants. Most of these variants (4.2%, 8/191) were identified in the moderate to high penetrance genes *BRCA2*, *MSH6*, *CHEK2* and *ATM*. The detection rate in these core genes is comparable to previous reports^14,15^. We also detected a likely pathogenic variant in *MUTYH*, for which there is still insufficient evidence for a significant risk of BC.

Interpreting the pathogenicity significance of moderate- or low- penetrance variants is a challenge for current cancer risk management, counselling and treatment decision-making regarding patients and their families. In this regard, Lynch syndrome (LS) is an autosomal dominant condition caused by pathogenic variants in one of the MMR genes, which result in different cancer risks. *Path*_*MLH1* and *path*_*MSH2* variants are highly penetrant when comparing to *path_MSH6* and *path*_*PMS2* variants^44–46^. Regarding *BRCA* genes, we have recently described that the most frequent *path_BRCA1* variants in the Norwegian population had low penetrance in fertile ages, and lower than in carriers of infrequent *path_BRCA1* variants^47^. The relative risk of developing BC has been described for truncating variants in *BRCA2* (relative risk: 11.7), while *ATM* and *CHEK2* truncating variants have relative risks of 2.8 (2.2–3.7) and 3.0 (2.6–3.5), respectively^48^.

A recent evaluation of gene-disease associations using the ClinGen clinical validity framework revealed definitive assertion for *ATM* and *CHEK2* genes in breast cancer^49^. Our results are in agreement for the pathogenic variants found in the *ATM* (n = 3) and *CHEK2* (n = 1) genes which were found in phenocopies or familial BC cases^49^.

There is still no clear evidence of an association between BC and other DNA repair genes, such as *MLH1*, *MSH2*, *MSH6* and *MAP3K1* genes. An average moderate-risk colorectal screening based on family history has been suggested for individuals with a heterozygous germline *MUTYH* pathogenic variant^50,51^.

Approximately 25% of missense and nonsense variants disrupt mRNA splicing, and the understanding of functional splice variations may allow the (re)classification of VUS in CRC and other BC-associated genes. In a recent retrospective study, among variants initially classified as VUS, 7.7% were reclassified (91% were downgraded to class 1/2 and 9% were upgraded class 4/5) and 8.7% were upgraded to class 4/5^52^.

Out of the 87 variants of VUS, 11 were tested by a minigene splicing assay, out of which two (*ATM* c.3806A > G and *BUB1* c.677C > T) were demonstrated to exert an effect on splicing. The *ATM* c.3806A > G variant was identified in an early-onset BC patient (<50 years) with a strong BC, ovarian and colon cancer family history. Our minigene assay results suggested that this variant partially alters the splicing pattern of *ATM*, leading to the concomitant production of aberrant transcripts presenting in-frame deletions in exon 26 and a decreased expression of full-length transcripts carrying the c.3806A > G (p.Lys1269Arg) variant. Although these experimental findings agree with RNA-splicing-dedicated *in silico* predictions, and minigene results are often concordant with *in vivo* data^43,53,54^, it will be essential to confirm our experimental results by analyzing RNA from patient samples. However, no RNA from the peripheral blood nor from the tumor of this patient, nor the other patients, were available for analysis. Our IHC results suggest that *ATM* c.3806A > G is associated with tumor development in the index patient. However, segregation data may provide additional evidence to classify this variant.

There is no clinical significance reported for the *BUB1* c.677C > T variant. *BUB1* encodes a serine/threonine-protein kinase that plays a central role in mitosis^55^, and pathogenic variants in this gene have been associated with aneuploidy and several forms of cancer. However, a recent study does not support the inclusion of *BUB1* and *BUB3* testing in routine genetic diagnostics of familial CRC and polyposis^56^. Our minigene assay showed a moderate increase in exon skipping due to the *BUB1* c.677C > T variant, that remains classified as a VUS.

Information about *ATM* c.3806A > G and *BUB1* c.677C > T variants in the spectrum of BC studies using NGS panel testing is currently scarce. In this scenario, not only functional or protein assays, but also co-segregation studies, will be essential for understanding whether the VUS analyzed in this work have causal or modifying effects, or otherwise are non-pathogenic. Importantly, there is no systematic classification for most of the genetic variants found by NGS, including the *ATM* c.3806A > G and *BUB1* c.677C > T identified in this study, and, in more general terms, the impact of pathogenic variants in low- to moderate-penetrance genes is not fully understood with respect to clinical management. Most of the VUS may in the future be reclassified as deleterious or benign, but in the meantime, they cannot be used to make clinical decisions^22^.

There is a need to understand and quantify the cancer risk of pathogenic and currently uncertain variants found in moderate- or low- penetrance genes. A quantitative approach to prioritize missense substitutions with high probabilities of pathogenicity has been suggested for the *ATM*, *CHEK2* and *NBN* genes^57^. However, there is still a high number of novel variants with an unknown significance of pathogenicity identified in cancer patients with family history awaiting classification. The evaluation of clinical, family parameters, functional assay and IHC analyses undertaken in this study may allow to refine the clinical classification of these variants. To the latter end, broad collaborations to compile data from different sources are needed to arrive at conclusions, and, we here contribute our results towards that end.

## Conclusion

Five percent of 191 families initially tested by clinical diagnostic settings, were found to carry pathogenic variants in the *ATM*, *MSH6*, *BRCA2*, *MUTYH* and *CHEK2* genes, when using a 44-gene panel. We further provided functional testing and protein characterization in addition to clinical and family history information.

Our study contributes to understanding and interpreting variants in moderate- or low-penetrance genes which are not routinely tested in the clinical genetic settings and may have a direct impact on providing an appropriate genetic counseling and clinical management for individuals and their relatives who carry these variants.

## Supplementary information

Suppl Table 1

Supplementary Table 2

Supplementary Table 3
