# Supplementary material for: Results of multigene panel testing in familial cancer cases without genetic cause demonstrated by single gene testing
Source: Sci Rep. 2019 Dec 6;9:18555. doi: 10.1038/s41598-019-54517-z (PMC6898579; doi:10.1038/s41598-019-54517-z)
Supplement: Supplementary file 1 — Suppl Table 1 [file 41598_2019_54517_MOESM1_ESM.pdf]

Results of multigene panel testing in familial cancer cases without genetic cause demonstrated by single gene testing

Mev Dominguez-Valentin1, Sigve Nakken1,14, Hélène Tubeuf2,3, Daniel Vodak1, Per Olaf Ekstrøm1, Anke M. Nissen4,5, Monika Morak4,5, Elke Holinski-Feder4,5, Arild Holth6, Gabriel Capella7, Ben Davidson6,8, D. Gareth Evans9,10, Alexandra Martins2, Pål Møller1,11,12, Eivind Hovig1,13

Supplementary Table 1. Illumina TruSight target design information for the 44 cancer gene panel including the target region region, chromosom start and stop coordinate, lenght for each targeted gene region, SNP count and the coverage of the design for each target gene region.

Target Gene Region	Chromosome	Start Coordinate	Stop Coordinate	Length	Amplicons	SNP Count	Coverage
APC	5	112173225	112181961	8737	32	34	100
APC	5	112073531	112074182	652	3	2	100
APC_Ex_2021042 + Ex_2018339 +Exon_2019565	5	112162805	112164669	1865	4	0	59
APC_Exon_2017752	5	112116487	112116600	114	1	0	100
APC_Exon_2017753	5	112170648	112170862	215	1	0	100
APC_Exon_2018122	5	112102023	112102107	85	1	2	100
APC_Exon_2018123	5	112136976	112137080	105	1	6	100
APC_Exon_2018179	5	112102886	112103087	202	1	0	100
APC_Exon_2018180 + Exon_2018180	5	112151042	112151440	399	2	0	100
APC_Exon_2018864	5	112157593	112157688	96	1	0	100
APC_Exon_2019978	5	112111326	112111434	109	1	0	100
APC_Exon_2020381	5	112154663	112155041	379	2	0	100
APC_Exon_2021041	5	112043202	112043579	378	2	2	100
APC_Exon_2265614	5	112090570	112090722	153	1	12	100
APC_Exon_2267008	5	112128143	112128226	84	1	4	100
ATM	11	108143234	108143604	371	2	2	100
ATM	11	108235784	108239851	4068	11	16	77
ATM	11	108202146	108202789	644	2	0	90
ATM	11	108098297	108098640	344	1	4	100
ATM	11	108186525	108186865	341	2	4	100
ATM	11	108159679	108160553	875	3	2	100

ATM	11	108141725	108142249	525	1	0	47
ATM + Exon_1950810	11	108163321	108164254	934	4	2	100
ATM + Exon_1955421	11	108196012	108197002	991	4	10	100
ATM_Exon_1950050	11	108099855	108100100	246	1	0	100
ATM_Exon_1950051	11	108119610	108119879	270	1	0	100
ATM_Exon_1950553	11	108158327	108158442	116	1	2	100
ATM_Exon_1950554	11	108165654	108165786	133	1	2	100
ATM_Exon_1950567	11	108115515	108115753	239	1	6	100
ATM_Exon_1950568	11	108117691	108117854	164	1	0	100
ATM_Exon_1950569	11	108122564	108122758	195	1	0	100
ATM_Exon_1950570	11	108123544	108123639	96	1	0	100
ATM_Exon_1950572	11	108183138	108183225	88	1	0	100
ATM_Exon_1950725	11	108175402	108175579	178	1	0	100
ATM_Exon_1950811	11	108190681	108190785	105	1	2	100
ATM_Exon_1950812 + Exon_1950812	11	108191928	108192247	320	1	0	100
ATM_Exon_1950813	11	108198372	108198485	114	1	0	100
ATM_Exon_1950901	11	108128208	108128333	126	1	0	100
ATM_Exon_1950903	11	108168014	108168109	96	1	0	100
ATM_Exon_1950904	11	108224493	108224607	115	1	2	100
ATM_Exon_1950977	11	108151722	108151895	174	1	0	100
ATM_Exon_1950978	11	108153437	108153606	170	1	4	100
ATM_Exon_1950979	11	108170441	108170612	172	1	0	100
ATM_Exon_1950980	11	108200941	108201148	208	1	0	100
ATM_Exon_1950981	11	108216470	108216635	166	1	0	100
ATM_Exon_1951064	11	108121428	108121799	372	2	2	100
ATM_Exon_1951065	11	108124541	108124766	226	1	4	100
ATM_Exon_1951146	11	108106397	108106561	165	1	2	100
ATM_Exon_1951147	11	108139137	108139336	200	1	14	100
ATM_Exon_1951149	11	108172375	108172516	142	1	0	100
ATM_Exon_1951150	11	108178624	108178711	88	1	0	100
ATM_Exon_1951240	11	108218006	108218092	87	1	2	100
ATM_Exon_1952318	11	108150218	108150335	118	1	2	100

ATM_Exon_1952319	11	108173580	108173756	177	1	0	100
ATM_Exon_1952651	11	108204613	108204695	83	1	0	100
ATM_Exon_1952652	11	108205696	108205836	141	1	0	100
ATM_Exon_1952653	11	108225538	108225601	64	1	2	100
ATM_Exon_1952908 + Exon_1952908	11	108129563	108129952	390	2	2	100
ATM_Exon_1952909	11	108154954	108155200	247	1	4	100
ATM_Exon_1954841	11	108213949	108214098	150	1	0	100
ATM_Exon_1955415	11	108093559	108093913	355	2	2	100
ATM_Exon_1955416	11	108114680	108114845	166	1	0	100
ATM_Exon_1955417	11	108126842	108127100	259	1	0	100
ATM_Exon_1955418	11	108137898	108138069	172	1	0	100
ATM_Exon_1955420	11	108187970	108188340	371	2	4	100
ATM_Exon_1955422	11	108199617	108200128	512	2	0	100
ATM_Exon_1955423	11	108203489	108203627	139	1	0	100
ATM_Exon_1955424	11	108206572	108206688	117	1	0	100
ATM_Exon_1955727	11	108180887	108181042	156	1	2	100
AXIN2	17	63532413	63534486	2074	7	10	90
AXIN2_Exon_2118232	17	63531744	63531839	96	1	0	100
AXIN2_Exon_2118319	17	63530030	63530197	168	1	0	100
AXIN2_Exon_2118418	17	63557568	63557740	173	1	2	100
AXIN2_Exon_2118501	17	63545638	63545778	141	1	0	100
AXIN2_Exon_2118654	17	63553924	63554854	931	4	0	100
AXIN2_Exon_2118831	17	63524683	63526220	1538	6	0	100
AXIN2_Exon_2122329	17	63537573	63537675	103	1	0	100
BAP1	3	52435000	52437935	2936	9	6	87
BAP1	3	52439101	52442647	3547	13	12	100
BAP1	3	52443545	52444034	490	2	0	100
BAP1_Exon_2195407	3	52438469	52438602	134	1	0	100
BARD1_Exon_1852481	2	215674136	215674428	293	1	0	100
BARD1_Exon_1852559	2	215632206	215632378	173	1	4	100
BARD1_Exon_1852560 + Exon_1852560	2	215633806	215634186	381	1	2	65
BARD1_Exon_1853138	2	215595135	215595232	98	1	2	100

BARD1_Exon_1853139	2	215657021	215657169	149	1	0	100
BARD1_Exon_1854769	2	215610446	215610578	133	1	0	100
BARD1_Exon_1854892	2	215617171	215617279	109	1	0	100
BARD1_Exon_1855753	2	215645284	215646233	950	4	4	100
BARD1_Exon_1855754	2	215661785	215661841	57	1	0	100
BARD1_Exon_1856283	2	215609791	215609883	93	1	0	100
BARD1_Exon_1857115	2	215593275	215593732	458	2	10	100
BMPR1A	10	88683108	88684970	1863	7	4	100
BMPR1A	10	88659526	88659908	383	2	2	100
BMPR1A_Exon_2246225	10	88635624	88635842	219	1	0	100
BMPR1A_Exon_2246636	10	88678929	88679226	298	1	0	100
BMPR1A_Exon_2246727+ Exon_2246727	10	88516134	88516835	702	2	2	56
BMPR1A_Exon_2246728	10	88649819	88649981	163	1	2	100
BMPR1A_Exon_2246729	10	88671997	88672141	145	1	0	100
BMPR1A_Exon_2247064	10	88598622	88598736	115	1	0	100
BMPR1A_Exon_2247066	10	88681277	88681452	176	1	0	100
BMPR1A_Exon_2248431	10	88651884	88651986	103	1	0	100
BMPR1A_Exon_2248764	10	88676891	88677083	193	1	0	100
BRCA1	17	41251717	41251972	256	1	0	100
BRCA1	17	41277174	41277525	352	2	2	100
BRCA1	17	41228480	41228656	177	1	0	100
BRCA1	17	41258448	41258575	128	1	0	100
BRCA1	17	41242886	41246952	4067	15	454	100
BRCA1_Exon_1950723	17	41215891	41215968	78	1	0	100
BRCA1_Exon_1950887	17	41219625	41219712	88	1	2	100
BRCA1_Exon_1950888	17	41256885	41256973	89	1	6	100
BRCA1_Exon_1951427	17	41215350	41215390	41	1	2	100
BRCA1_Exon_1951909	17	41199660	41199720	61	1	0	100
BRCA1_Exon_1951910+Exon_1951910	17	41247763	41248039	277	1	2	100
BRCA1_Exon_1951911	17	41255644	41256409	766	2	22	76
BRCA1_Exon_1952422+Exon_1952422	17	41208919	41209302	384	1	0	66
BRCA1_Exon_1952502	17	41196262	41197869	1608	3	12	55

BRCA1_Exon_1952503	17	41222945	41223255	311	1	0	100
BRCA1_Exon_1952504	17	41234421	41234592	172	1	2	100
BRCA1_Exon_1954922	17	41226348	41226538	191	1	14	100
BRCA1_Exon_1954924	17	41267743	41267796	54	1	0	100
BRCA1_Exon_1955584 + Exon_1955584	17	41249111	41249456	346	1	0	100
BRCA1_Exon_1956572	17	41276034	41276132	99	1	0	100
BRCA1_Exon_1956968	17	41203080	41203134	55	1	4	100
BRCA1_Exon_2112460	17	41201138	41201211	74	1	4	100
BRCA1_Exon_2116381	17	41230847	41231853	1007	1	0	20
BRCA2	13	32953429	32954307	879	3	50	100
BRCA2	13	32936635	32937695	1061	4	46	100
BRCA2	13	32944514	32945262	749	2	26	66
BRCA2	13	32900163	32900825	663	3	18	100
BRCA2_Ex_2150341+Ex_2150340+Ex_2155357	13	32903096	32907574	4479	14	130	89
BRCA2_Exon_2150343	13	32968826	32969070	245	1	0	100
BRCA2_Exon_2150518	13	32928998	32929425	428	2	16	100
BRCA2_Exon_2150520	13	32950807	32950928	122	1	0	100
BRCA2_Exon_2150521	13	32972299	32973809	1511	4	22	70
BRCA2_Exon_2150617	13	32910352	32915383	5032	18	394	96
BRCA2_Exon_2150697	13	32899213	32899321	109	1	0	100
BRCA2_Exon_2150699	13	32920964	32921033	70	1	4	100
BRCA2_Exon_2150780	13	32889617	32889804	188	1	2	100
BRCA2_Exon_2150781	13	32918695	32918790	96	1	0	100
BRCA2_Exon_2150782	13	32931879	32932066	188	1	0	100
BRCA2_Exon_2150953	13	32930565	32930746	182	1	2	100
BRCA2_Exon_2150954	13	32971035	32971181	147	1	0	100
BRCA2_Exon_2152552	13	32893214	32893462	249	1	0	100
BRCA2_Exon_2152622	13	32890559	32890664	106	1	0	100
BRIP1	17	59857360	59858872	1513	4	8	78
BRIP1_Exon_2135398	17	59793312	59793424	113	1	0	100
BRIP1_Exon_2135814	17	59756547	59761501	4955	13	8	74
BRIP1_Exon_2135815	17	59937157	59937268	112	1	0	100

BRIP1_Exon_2135897	17	59924462	59924581	120	1	2	100
BRIP1_Exon_2135986	17	59938808	59938930	123	1	0	100
BRIP1_Exon_2136079	17	59870958	59871090	133	1	0	100
BRIP1_Exon_2136230	17	59885728	59886218	491	2	4	100
BRIP1_Exon_2136652	17	59821640	59822026	387	2	0	100
BRIP1_Exon_2136653	17	59940645	59940920	276	1	4	100
BRIP1_Exon_2137000	17	59876461	59876660	200	1	0	100
BRIP1_Exon_2137154	17	59763197	59763526	330	1	0	100
BRIP1_Exon_2138318	17	59770791	59770873	83	1	0	100
BRIP1_Exon_2138319	17	59853762	59853923	162	1	0	100
BRIP1_Exon_2138320	17	59934419	59934592	174	1	0	100
BRIP1_Exon_2138681	17	59820374	59820495	122	1	0	100
BRIP1_Exon_2138682	17	59926490	59926617	128	1	2	100
BRIP1_Exon_2138836	17	59861631	59861785	155	1	0	100
BRIP1_Exon_2138837	17	59878614	59878835	222	1	0	100
BUB1	2	111415962	111416344	383	2	0	100
BUB1	2	111425073	111425451	379	2	0	100
BUB1	2	111414588	111415247	660	3	2	100
BUB1	2	111431638	111431967	330	2	2	100
BUB1	2	111398586	111399836	1251	5	4	100
BUB1_Exon_2223146	2	111427030	111427130	101	1	0	100
BUB1_Exon_2223304	2	111413316	111413493	178	1	0	100
BUB1_Exon_2223408	2	111397319	111397425	107	1	0	100
BUB1_Exon_2223818	2	111406646	111407128	483	1	2	53
BUB1_Exon_2223819	2	111430095	111430505	411	2	4	100
BUB1_Exon_2224432	2	111395409	111395736	328	1	2	100
BUB1_Exon_2224433	2	111408123	111408361	239	1	2	100
BUB1_Exon_2224943	2	111428102	111428145	44	1	0	100
BUB1_Exon_2225097	2	111435497	111435734	238	1	2	100
BUB1_Exon_2225472 + Exon_2225472	2	111417405	111417763	359	2	0	100
BUB1_Exon_2225473	2	111423840	111423991	152	1	0	100
BUB1_Exon_2225818	2	111419159	111419418	260	1	0	100

BUB1_Exon_2225910	2	111410827	111411225	399	2	2	100
BUB3	10	124913735	124914653	919	4	4	100
BUB3	10	124921727	124922369	643	3	0	100
BUB3	10	124923311	124924911	1601	6	2	100
BUB3_Exon_2001431	10	124917245	124917396	152	1	0	100
BUB3_Exon_2001432	10	124919923	124920081	159	1	0	100
BUB3_Exon_2003522	10	124915174	124915243	70	1	2	100
CDH1	16	68842302	68842776	475	2	4	100
CDH1	16	68845562	68846191	630	3	8	100
CDH1_Exon_1869260	16	68844100	68844244	145	1	0	100
CDH1_Exon_1869337	16	68835573	68835796	224	1	0	100
CDH1_Exon_1869454	16	68847216	68847398	183	1	0	100
CDH1_Exon_1869455	16	68857302	68857529	228	1	0	100
CDH1_Exon_1869695	16	68855904	68856128	225	1	4	100
CDH1_Exon_1870347	16	68867193	68869444	2252	7	10	90
CDH1_Exon_1870537	16	68771195	68771366	172	1	0	100
CDH1_Exon_1872690	16	68772200	68772314	115	1	0	100
CDH1_Exon_1872691	16	68849418	68849662	245	1	0	100
CDH1_Exon_1872692	16	68862077	68862207	131	1	0	100
CDH1_Exon_1873486	16	68863507	68863750	244	1	0	100
CDH1_Exon_1874308	16	68853183	68853328	146	1	4	100
CDKN2A	9	21974378	21975157	780	3	4	100
CDKN2A	9	21967726	21968795	1070	4	6	100
CDKN2A_Exon_1891068	9	21970901	21971207	307	1	0	100
CDKN2A_Exon_2259042	9	21994138	21994490	353	1	0	100
CDX2_Exon_2218652	13	28536278	28537506	1229	5	6	100
CDX2_Exon_2219743	13	28542603	28543317	715	3	2	100
CDX2_Exon_2220188	13	28539007	28539152	146	1	0	100
CHEK1	11	125499102	125499380	279	1	2	100
CHEK1	11	125525095	125525665	571	2	0	100
CHEK1	11	125513662	125514563	902	2	0	77
CHEK1	11	125495011	125496753	1743	7	10	100

CHEK1_Exon_2008398	11	125523541	125523842	302	1	0	100
CHEK1_Exon_2010334	11	125504725	125505559	835	3	4	100
CHEK1_Exon_2010493	11	125503058	125503246	189	1	0	100
CHEK1_Exon_2010584	11	125497502	125497725	224	1	2	100
CHEK1_Exon_2011355	11	125507344	125507439	96	1	0	100
CHEK1_Exon_2190773	11	125545117	125546895	1779	5	6	73
CHEK2	22	29120840	29121480	641	1	2	54
CHEK2	22	29091090	29091886	797	2	4	67
CHEK2_Exon_1951634	22	29130391	29130715	325	1	0	100
CHEK2_Exon_1952800	22	29083681	29084024	344	1	2	100
CHEK2_Exon_1952803	22	29137757	29137822	66	1	0	100
CHEK2_Exon_1953091	22	29085123	29085203	81	1	0	100
CHEK2_Exon_1953092	22	29099493	29099554	62	1	0	100
CHEK2_Exon_1953597	22	29090020	29090105	86	1	2	100
CHEK2_Exon_1953598	22	29115383	29115473	91	1	0	100
CHEK2_Exon_1953909	22	29105994	29106047	54	1	0	100
CHEK2_Exon_1953993	22	29107897	29108005	109	1	0	100
CHEK2_Exon_1954256	22	29095826	29095925	100	1	0	100
CHEK2_Exon_1956164	22	29092889	29092975	87	1	4	100
CHEK2_Exon_2007208 + Exon_2007208	22	29126145	29126564	420	0	0	0
CTNNB1	3	41265487	41267377	1891	7	94	100
CTNNB1	3	41274807	41275813	1007	4	4	100
CTNNB1	3	41280600	41281964	1365	5	14	100
CTNNB1	3	41277815	41278225	411	2	90	100
CTNNB1_Exon_2004993	3	41240942	41241161	220	1	0	100
CTNNB1_Exon_2006997	3	41277215	41277334	120	1	0	100
CTNNB1_Exon_2007100	3	41279507	41279567	61	1	2	100
CTNNB1_Exon_2007236	3	41268699	41268843	145	1	0	100
DVL2	17	7128636	7131388	2753	9	8	92
DVL2	17	7132263	7134141	1879	6	0	90
DVL2_Exon_2056452	17	7137388	7137863	476	2	0	100
EPCAM	2	47600577	47601212	636	3	4	100

EPCAM_Exon_1879191	2	47606858	47607158	301	1	2	100
EPCAM_Exon_1879192	2	47612305	47612349	45	1	0	100
EPCAM_Exon_1879334	2	47596287	47596720	434	2	0	100
EPCAM_Exon_1879450 + Exon_1879450	2	47604053	47604316	264	1	0	100
EPCAM_Exon_1879775	2	47606092	47606193	102	1	0	100
EPCAM_Exon_1882750	2	47613661	47614217	557	1	2	50
EPCAM_Exon_1884776	2	47602373	47602438	66	1	2	100
FAM175A_E_2122351+_E_2122352+_E_2122353	4	84388607	84391549	2943	7	0	69
FAM175A_Ex_2120375+_2123427++FAM175A_Ex	4	84382094	84384911	2818	8	8	84
FAM175A_Exon_2120536	4	84397796	84397832	37	1	0	100
FAM175A_Exon_2120640	4	84403307	84403397	91	1	2	100
FAM175A_Exon_2122137	4	84406139	84406290	152	1	0	100
FAM175A_Exon_2122991	4	84393375	84393441	67	1	0	100
FAM175B_Exon_2061711+ Exon_2061711	10	126494938	126496679	1742	2	0	40
FAM175B_Exon_2061712	10	126505145	126505181	37	1	0	100
FAM175B_Exon_2061713	10	126519918	126520032	115	1	0	100
FAM175B_Exon_2062054	10	126517325	126517444	120	1	0	100
FAM175B_Exon_2062139	10	126523071	126525239	2169	8	12	100
FAM175B_Exon_2062220	10	126490354	126490470	117	1	0	100
FAM175B_Exon_2063807	10	126507856	126508212	357	2	0	100
FAM175B_Exon_2063994	10	126515064	126515454	391	2	2	100
FAM175B_Exon_2063995	10	126517962	126518046	85	1	0	100
FGFR2	10	123237819	123239560	1742	7	10	100
FGFR2	10	123353198	123353797	600	3	2	100
FGFR2	10	123274606	123274858	253	1	0	100
FGFR2 + Exon_1894461 + Exon_1894461	10	123246843	123247727	885	3	24	91
FGFR2_Exon_1893695	10	123298106	123298229	124	1	2	100
FGFR2_Exon_1893779	10	123357476	123357972	497	2	2	100
FGFR2_Exon_1894102	10	123244909	123245046	138	1	0	100
FGFR2_Exon_1894445	10	123258009	123258119	111	1	2	100
FGFR2_Exon_1894462	10	123279493	123279683	191	1	0	100
FGFR2_Exon_1895058	10	123241367	123241691	325	1	6	100

FGFR2_Exon_1895059	10	123263304	123263455	152	1	0	100
FGFR2_Exon_1895224	10	123243212	123243317	106	1	0	100
FGFR2_Exon_1895225 + Exon_1895225	10	123310254	123311223	970	3	8	79
FGFR2_Exon_1896050	10	123324016	123324093	78	1	2	100
FGFR2_Exon_1896126	10	123260340	123260461	122	1	2	100
FGFR2_Exon_1896127	10	123278196	123278343	148	1	0	100
FGFR2_Exon_1896380	10	123256046	123256236	191	1	0	100
FGFR2_Exon_2008715	10	123276833	123276977	145	1	0	100
FGFR2_Exon_2141967	10	123324952	123325218	267	1	0	100
FGFR2_Exon_2284748	10	123355991	123356159	169	1	2	100
MAP3K1	5	56160486	56161879	1394	3	0	70
MAP3K1	5	56168443	56168857	415	2	2	100
MAP3K1	5	56176513	56178718	2206	8	38	97
MAP3K1_Exon_2290037	5	56181759	56181890	132	1	0	100
MAP3K1_Exon_2290118	5	56110900	56111882	983	3	2	71
MAP3K1_Exon_2290119	5	56189358	56191978	2621	9	4	100
MAP3K1_Exon_2290279	5	56167737	56167858	122	1	0	100
MAP3K1_Exon_2290282	5	56184053	56184184	132	1	0	100
MAP3K1_Exon_2290825	5	56152427	56152577	151	1	2	100
MAP3K1_Exon_2290826	5	56170859	56171137	279	1	2	100
MAP3K1_Exon_2291452	5	56174807	56174928	122	1	0	100
MAP3K1_Exon_2291454	5	56179354	56179506	153	1	0	100
MAP3K1_Exon_2291976	5	56180491	56180653	163	1	0	100
MAP3K1_Exon_2292354	5	56155542	56155742	201	1	0	100
MAP3K1_Exon_2292355	5	56183205	56183347	143	1	0	100
MLH1	3	37053286	37053615	330	1	0	100
MLH1	3	37034816	37035406	591	3	6	100
MLH1	3	37042421	37042569	149	1	0	100
MLH1	3	37038049	37038260	212	1	0	100
MLH1	3	37089933	37090583	651	3	214	100
MLH1_Exon_1914206	3	37067128	37067498	371	2	18	100
MLH1_Exon_1914285	3	37058997	37059090	94	1	0	100

MLH1_Exon_1914378	3	37045892	37045965	74	1	0	100
MLH1_Exon_1914379	3	37055923	37056035	113	1	0	100
MLH1_Exon_1914530	3	37048482	37048554	73	1	2	100
MLH1_Exon_1914610	3	37061801	37061954	154	1	4	100
MLH1_Exon_1915098	3	37083700	37083900	201	1	0	100
MLH1_Exon_1915412	3	37091927	37092387	461	2	54	100
MLH1_Exon_1916639	3	37070275	37070423	149	1	0	100
MLH1_Exon_1917495	3	37081677	37081785	109	1	4	100
MLH1_Exon_1917611+ Exon_1917611	3	37088910	37089274	365	2	118	100
MLH1_Exon_2038480	3	37050305	37050396	92	1	2	100
MRE11A	11	94226825	94227065	241	1	4	100
MRE11A_Exon_2236473	11	94170343	94170401	59	1	2	100
MRE11A_Exon_2236474	11	94194102	94194202	101	1	0	100
MRE11A_Exon_2236475	11	94204740	94204925	186	1	0	100
MRE11A_Exon_2236553	11	94180385	94180604	220	1	0	100
MRE11A_Exon_2236637	11	94192574	94192747	174	1	0	100
MRE11A_Exon_2236638	11	94219090	94219250	161	1	0	100
MRE11A_Exon_2236821	11	94189442	94189504	63	1	2	100
MRE11A_Exon_2236822	11	94197279	94197405	127	1	2	100
MRE11A_Exon_2236823	11	94223999	94224131	133	1	0	100
MRE11A_Exon_2237151	11	94163077	94163152	76	1	0	100
MRE11A_Exon_2237152	11	94200979	94201059	81	1	0	100
MRE11A_Exon_2237153	11	94212840	94212927	88	1	2	100
MRE11A_Exon_2237222	11	94150469	94153347	2879	10	6	93
MRE11A_Exon_2238755	11	94168998	94169065	68	1	4	100
MRE11A_Exon_2238756	11	94211901	94212042	142	1	2	100
MRE11A_Exon_2238915	11	94225948	94226072	125	1	2	100
MRE11A_Exon_2238997	11	94209405	94209619	215	1	2	100
MRE11A_Exon_2239321	11	94203637	94203808	172	1	0	100
MRE11A_Exon_2277593	11	94178850	94179200	351	1	2	100
MSH2_Exon_1988795	2	47690170	47690293	124	1	2	100
MSH2_Exon_1989651	2	47672687	47672796	110	1	0	100

MSH2_Exon_1990005	2	47703506	47703710	205	1	4	100
MSH2_Exon_1990006	2	47705411	47705658	248	1	0	100
MSH2_Exon_1990172	2	47637233	47637511	279	1	2	100
MSH2_Exon_1990173	2	47698054	47698251	198	1	56	100
MSH2_Exon_1990613	2	47641408	47641557	150	1	10	100
MSH2_Exon_1990614	2	47693747	47693997	251	1	54	100
MSH2_Exon_1991019	2	47639503	47639749	247	1	0	100
MSH2_Exon_1991020	2	47709918	47710360	443	2	2	100
MSH2_Exon_1991849	2	47635490	47635855	366	2	2	100
MSH2_Exon_1992093	2	47702164	47702409	246	1	0	100
MSH2_Exon_1992094	2	47707835	47708010	176	1	0	100
MSH2_Exon_1993122	2	47630263	47630541	279	1	2	100
MSH2_Exon_1993123	2	47643435	47643568	134	1	0	100
MSH2_Exon_1993124	2	47656881	47657080	200	1	6	100
MSH6	2	48032732	48034117	1386	5	192	100
MSH6_Exon_2112304	2	48022983	48023252	270	1	2	100
MSH6_Exon_2112942	2	48018066	48018262	197	1	4	100
MSH6_Exon_2113179	2	48030559	48030824	266	1	2	100
MSH6_Exon_2114169	2	48010171	48010682	512	2	6	100
MSH6_Exon_2115373	2	48025700	48028344	2645	10	72	100
MSH6_Exon_2116506 + Exon_2116506	2	48031949	48032266	318	1	6	100
MUTYH	1	45805546	45806167	622	3	6	100
MUTYH	1	45796829	45799300	2472	7	2	82
MUTYH_Exon_1834267+ Exon_1832588	1	45794426	45796339	1914	3	0	46
MUTYH_Exon_1834268	1	45800063	45800183	121	1	2	100
NBN	8	90992937	90993776	840	3	2	100
NBN_Exon_2131142	8	90994950	90995083	134	1	0	100
NBN_Exon_2132260	8	90949254	90949303	50	1	0	100
NBN_Exon_2132261	8	90958368	90958523	156	1	2	100
NBN_Exon_2132262	8	90990448	90990551	104	1	0	100
NBN_Exon_2132382	8	90955481	90955594	114	1	0	100
NBN_Exon_2132383	8	90965472	90965919	448	2	0	100

NBN_Exon_2132384	8	90983401	90983518	118	1	0	100
NBN_Exon_2132523	8	90976638	90976735	98	1	0	100
NBN_Exon_2132524	8	90982592	90982785	194	1	4	100
NBN_Exon_2132554	8	90967511	90967783	273	1	4	100
NBN_Exon_2133030	8	90960052	90960120	69	1	4	100
NBN_Exon_2133083	8	90996703	90996949	247	1	54	100
NBN_Exon_2133408	8	90945564	90947840	2277	9	16	100
NBN_Exon_2133850	8	90970953	90971082	130	1	4	100
NOTCH3	19	15295081	15296515	1435	4	2	66
NOTCH3	19	15302210	15303355	1146	2	0	59
NOTCH3	19	15299775	15300264	490	1	2	55
NOTCH3	19	15276154	15276927	774	2	2	76
NOTCH3	19	15297664	15298174	511	2	6	100
NOTCH3	19	15298667	15299184	518	2	2	100
NOTCH3	19	15280872	15281661	790	3	2	100
NOTCH3_Exon_2068972	19	15273176	15273473	298	1	0	100
NOTCH3_Exon_2069479	19	15278010	15278272	263	1	0	100
NOTCH3_Exon_2070761	19	15270444	15272525	2082	8	8	100
NOTCH3_Exon_2070762	19	15288336	15288901	566	2	0	100
NOTCH3_Exon_2070928	19	15284879	15285211	333	1	4	100
NOTCH3_Exon_2073214	19	15311599	15311792	194	1	0	100
NOTCH3_Exon_2073517	19	15308311	15308389	79	1	0	100
NOTCH3	19	15289509	15292637	3129	8	2	71
PALB2	16	23649146	23649475	330	1	0	100
PALB2	16	23640500	23641815	1316	5	0	100
PALB2_Exon_2281839 + Exon_2281839	16	23634190	23634551	362	1	0	72
PALB2_Exon_2281999	16	23625325	23625412	88	1	0	100
PALB2_Exon_2282000	16	23635330	23635415	86	1	0	100
PALB2_Exon_2282424	16	23646183	23647655	1473	6	10	100
PALB2_Exon_2282606	16	23652431	23652678	248	1	0	100
PALB2_Exon_2282953	16	23614483	23614990	508	2	2	100
PALB2_Exon_2282954	16	23619185	23619333	149	1	0	100

PALB2_Exon_2282955	16	23632683	23632799	117	1	0	100
PALB2_Exon_2282956	16	23637557	23637718	162	1	0	100
PMS2	7	6043296	6043714	419	2	2	100
PMS2	7	6045498	6045687	190	1	8	100
PMS2_Exon_1914600	7	6026390	6027251	862	2	14	77
PMS2_Exon_1915021	7	603127	603794	668	3	0	100
PMS2_Exon_1915022	7	6038739	6038906	168	1	2	100
PMS2_Exon_1915512	7	6035165	6035264	100	1	2	100
PMS2_Exon_1915655	7	6048628	6048737	110	1	2	100
PMS2_Exon_1915939	7	6012870	6013173	304	1	0	100
PMS2_Exon_1916560 + Exon_1916560	7	6022284	6022719	436	0	0	0
PMS2_Exon_1916715	7	6036907	6037104	198	1	0	100
PMS2_Exon_1917092	7	6042084	6042267	184	1	2	100
PMS2_Exon_1917948 + Exon_1917948	7	6029281	6029736	456	2	2	100
POLD1	19	50905010	50906879	1870	5	12	78
POLD1	19	50909414	50910697	1284	3	2	63
POLD1	19	50902083	50902766	684	2	0	74
POLD1	19	50916658	50917161	504	2	4	100
POLD1	19	50912017	50912948	932	4	4	100
POLD1	19	50918047	50919108	1062	4	2	100
POLD1	19	50919628	50920551	924	3	6	88
POLD1_Exon_2136589	19	50887593	50887647	55	1	0	100
POLD1_Exon_2136948	19	50921049	50921321	273	1	0	100
POLE	12	133218208	133220588	2381	9	8	100
POLE	12	133202206	133202928	723	3	6	100
POLE	12	133237530	133238295	766	3	4	100
POLE	12	133200323	133201605	1283	5	8	100
POLE	12	133253924	133254330	407	1	0	60
POLE	12	133208876	133209406	531	2	0	100
POLE	12	133256058	133257890	1833	6	2	88
POLE	12	133251959	133253264	1306	5	2	100
POLE	12	133225490	133226500	1011	3	4	80

POLE	12	133233697	133234581	885	4	2	100
POLE	12	133244917	133245550	634	2	0	85
POLE	12	133248776	133250318	1543	6	8	100
POLE	12	133240515	133241123	609	3	4	100
POLE_Exon_1935810	12	133235881	133236095	215	1	4	100
POLE_Exon_1935813	12	133263840	133263945	106	1	0	100
POLE_Exon_1936221	12	133241888	133242036	149	1	2	100
POLE_Exon_1936222	12	133244039	133244284	246	1	0	100
POLE_Exon_1938399	12	133214600	133214725	126	1	0	100
POLE_Exon_1938654	12	133212478	133212610	133	1	2	100
POLE_Exon_1939374	12	133210772	133210964	193	1	2	100
POLE_Exon_1942079	12	133215711	133215884	174	1	0	100
PSMC3IP	17	40724304	40726253	1950	7	4	100
PSMC3IP	17	40729206	40729772	567	2	0	100
PTEN_Exon_1918508	10	89690803	89690846	44	1	0	100
PTEN_Exon_1918682	10	89685270	89685314	45	1	4	100
PTEN_Exon_1918683	10	89692770	89693008	239	1	0	100
PTEN_Exon_1919112	10	89720651	89720875	225	1	0	100
PTEN_Exon_1919190	10	89717610	89717776	167	1	0	100
PTEN_Exon_1919260	10	89711875	89712016	142	1	0	100
PTEN_Exon_1920862	10	89653782	89653866	85	1	0	100
PTEN_Exon_1921036	10	89725044	89728532	3489	13	24	100
PTEN_Exon_1921622	10	89623145	89624355	1211	5	4	100
RAD51C	17	56772267	56772720	454	2	0	100
RAD51C_Exon_2069743	17	56769963	56770149	187	1	0	100
RAD51C_Exon_2140538+ Exon_2140538	17	56795782	56800686	4905	10	4	59
RAD51C_Exon_2140807	17	56801401	56801461	61	1	2	100
RAD51C_Exon_2141215	17	56787220	56787351	132	1	0	100
RAD51C_Exon_2141298	17	56780557	56780690	134	1	0	100
RAD51C_Exon_2143632	17	56811479	56811692	214	1	0	100
RAD51C_Exon_2144159	17	56774054	56774220	167	1	0	100
RAD51C_Exon_2144440	17	56809845	56809905	61	1	0	100

RAD51L1_Exon_1842988 + Exon_1842988	14	68292081	68292394	314	1	0	100
RAD51L1_Exon_1842989	14	68878141	68878244	104	1	0	100
RAD51L1_Exon_1843075	14	68331720	68331856	137	1	2	100
RAD51L1_Exon_1843076	14	68352586	68352705	120	1	4	100
RAD51L1_Exon_1844046	14	68301797	68301913	117	1	0	100
RAD51L1_Exon_1845610	14	68286496	68286570	75	1	0	100
RAD51L1_Exon_1845611	14	68290259	68290344	86	1	0	100
RAD51L1_Exon_1845612	14	68353738	68353921	184	1	0	100
RAD51L1_Exon_1845613	14	68758601	68758697	97	1	2	100
RAD51L1_Exon_1845858	14	68934889	68934967	79	1	2	100
RAD51L1_Exon_1849196	14	68963841	68964598	758	3	2	100
RAD51L1_Exon_1943519	14	69061202	69062738	1537	5	4	86
RAD51L1_Exon_1946690	14	68944365	68944810	446	2	4	100
RAD51L3	17	33426786	33428409	1624	6	22	100
RAD51L3	17	33430248	33430588	341	1	2	100
RAD51L3	17	33433982	33434491	510	2	0	100
RAD51L3	17	33445495	33446913	1419	4	0	67
RAD51L3_Exon_1939576	17	33433405	33433500	96	1	0	100
RAD51L3_Exon_1988666	17	33443878	33444056	179	1	0	100
SMAD4	18	48584470	48584851	382	2	2	100
SMAD4	18	48575031	48575719	689	3	2	100
SMAD4_Exon_1928588	18	48573290	48573665	376	2	2	100
SMAD4_Exon_1928589	18	48593389	48593557	169	1	0	100
SMAD4_Exon_1928738	18	48586236	48586286	51	1	4	100
SMAD4_Exon_1928739	18	48604626	48611411	6786	22	6	89
SMAD4_Exon_1928910	18	48581101	48581413	313	2	0	100
SMAD4_Exon_1928911	18	48603008	48603146	139	1	2	100
SMAD4_Exon_1929403	18	48591793	48591976	184	1	2	100
SMAD4_Exon_1929587	18	48556583	48556993	411	2	0	100
STK11	19	1220347	1221364	1018	4	4	100
STK11_Exon_2002265	19	1227592	1228434	843	3	2	100
STK11_exon_2002659	19	1218366	1218549	184	1	54	100

STK11_Exon_2003080	19	1221948	1222005	58	1	0	100
STK11_Exon_2003761 + Exon_2003761	19	1219173	1219562	390	1	44	83
STK11_Exon_2004755	19	1226453	1226662	210	1	0	100
STK11_Exon_2006111	19	1222984	1223171	188	1	2	100
STK11_exon_2007676	19	1205798	1207202	1405	4	0	79
TP53	17	7576500	7577633	1134	2	4	57
TP53	17	7578152	7578836	685	2	6	62
TP53	17	7579287	7579965	679	3	102	100
TP53_Exon_1887091	17	7590695	7590863	169	1	0	100
TP53_Exon_1889309	17	7571580	7573058	1479	3	8	61
XRCC2_Exon_2186537	7	152357500	152357971	472	2	0	100
XRCC2_Exon_2186839	7	152373126	152373250	125	1	0	100
XRCC2_Exon_2187229	7	152345076	152346760	1685	4	6	70
XRCC3	14	104179187	104179330	144	1	0	100
XRCC3	14	104177345	104177929	585	2	0	70
XRCC3	14	104163929	104165938	2010	8	16	100
XRCC3_Exon_2172917	14	104181761	104181823	63	1	0	100
XRCC3_Exon_2173057	14	104174859	104174996	138	1	4	100
XRCC3_Exon_2173171	14	104169510	104169664	155	1	0	100
XRCC3_Exon_2176234	14	104173340	104173552	213	1	2	100
