# Supplementary material for: Results of multigene panel testing in familial cancer cases without genetic cause demonstrated by single gene testing
Source: Sci Rep. 2019 Dec 6;9:18555. doi: 10.1038/s41598-019-54517-z (PMC6898579; doi:10.1038/s41598-019-54517-z)
Supplement: Supplementary file 2 — Supplementary Table 2 [file 41598_2019_54517_MOESM2_ESM.docx]

Results of multigene panel testing in familial cancer cases without genetic cause demonstrated by single gene testing

Mev Dominguez-Valentin1, Sigve Nakken1,14, Hélène Tubeuf2,3, Daniel Vodak1, Per Olaf Ekstrøm1, Anke M. Nissen4,5, Monika Morak4,5, Elke Holinski-Feder4,5, Arild Holth6, Gabriel Capella7, Ben Davidson6,8, D. Gareth Evans9,10, Alexandra Martins2, Pål Møller1,11,12, Eivind Hovig1,13

| Purpose | Forward (F) or reverse (R) primers | |
| --- | --- | --- |
|  | Name | Sequence (5’-3’) |
| PCR (cloning, minigene preparation) | ATM_Ex26_InFus_Bam-F | AAGAAGTGCAGGATCCGAACTGACCCCAATTTGTTATTAAAGG |
|  | ATM_Ex26_InFus_Mlu-R | TCAAAACAAGACGCGTTGTAAACTGACAGCTAAAGTAAGGTATC |
|  | BUB1_Ex8_InFus_BamF | AAGAAGTGCAGGATCCAGCTATGGTTCTTCTCTACTGCTG |
|  | BUB1_Ex8_InFus_MluR | TCAAAACAAGACGCGTCTTGGACTAACTTGGCTGCTAC |
|  | CHEK1_Ex2_InFus_Bam-F | AAGAAGTGCAGGATCCGAGTGGCGATTGTGATTTACACG |
|  | CHEK1_Ex2_InFus_Mlu-R | TCAAAACAAGACGCGTGTGTCCCTTCCAGCTCTCTAC |
| Sequencing of minigene inserts | pCAS_Seq-F | GGGTCAATAGCAGTGAGAGG |
|  | pCAS_Seq-R | GCTCCATTTCACAGGTAGAGA |
| RTPCR and/or sequencing of RTPCR products | 6FAMpCASKO1F (5’fluo) | TGACGTCGCCGCCCATCAC |
|  | pCAS2R | ATTGGTTGTTGAGTTGGTTGTC |

Table S2. Primers used in the pCAS2 minigene splicing assay.
